# Autophagy in *Drosophila*: From Historical Studies to Current Knowledge

**DOI:** 10.1155/2014/273473

**Published:** 2014-05-18

**Authors:** Nitha C. Mulakkal, Peter Nagy, Szabolcs Takats, Radu Tusco, Gábor Juhász, Ioannis P. Nezis

**Affiliations:** ^1^School of Life Sciences, University of Warwick, Coventry CV4 7AL, UK; ^2^Department of Anatomy, Cell and Developmental Biology, Eötvös Loránd University, Budapest H-1117, Hungary

## Abstract

The discovery of evolutionarily conserved Atg genes required for autophagy in yeast truly revolutionized this research field and made it possible to carry out functional studies on model organisms. Insects including *Drosophila* are classical and still popular models to study autophagy, starting from the 1960s. This review aims to summarize past achievements and our current knowledge about the role and regulation of autophagy in *Drosophila*, with an outlook to yeast and mammals. The basic mechanisms of autophagy in fruit fly cells appear to be quite similar to other eukaryotes, and the role that this lysosomal self-degradation process plays in *Drosophila* models of various diseases already made it possible to recognize certain aspects of human pathologies. Future studies in this complete animal hold great promise for the better understanding of such processes and may also help finding new research avenues for the treatment of disorders with misregulated autophagy.

## 1. Introduction

Autophagy collectively refers to a group of intracellular degradation pathways that mediate the breakdown of intracellular material in lysosomes. This definition could as well include the endocytic downregulation of transmembrane proteins in the plasma membrane, but for historical and mechanistic reasons, that pathway is not considered to be part of autophagy. Different routes have evolved to solve the same topological issue; that is, cytoplasmic material including proteins, lipids, nucleic acids, and whole organelles including ER and mitochondria needs to be transported into the lumen of lysosomes. Three main subtypes are usually distinguished based on how cargo reaches the lysosome.

- During chaperone-mediated autophagy, a subset of individual proteins bearing a KFERQ amino acid sequence are unfolded and translocated across the lysosomal membrane through a channel consisting of LAMP2A proteins [1]. This pathway was described in cell-free systems and in cultured mammalian cells and its existence has not been shown in invertebrates yet.
- During microautophagy, invaginations of the lysosomal membrane pinch off portions of the cytoplasm. The resulting intraluminal vesicles are then broken down inside lysosomes. While the topology of this pathway resembles multivesicular endosome formation, genetic studies in yeast revealed that it requires a subset of the same genes that mediate the main, macroautophagic pathway. Although a morphological account of microautophagy is already found in a 1965 paper on the premetamorphotic insect fat body [2], this process is still difficult to study in metazoans, as no specific genes and reporters have been described yet. Thus, it is not discussed further here, and interested readers are suggested to consult a recent review on this topic [3].
- During macroautophagy, membrane cisterns called phagophores (also known as isolation membranes) assemble and capture cargo to be degraded. The resulting double-membrane autophagosomes then fuse with endosomes or lysosomes to give rise to amphisomes or autolysosomes, respectively. Autophagosome formation is enhanced in response to certain stress conditions such as starvation or during physiological changes triggered by hormonal cues [4, 5]. Thus, the degradative capacity of macroautophagy is the highest of the three pathways. As it is also the best studied route, it is usually simply referred to as autophagy, including the rest of this review.

## 2. Historical Early Studies

During the first 35–40 years of autophagy research, only a very limited methodological repertoire was available to study this process. The most commonly used technique was transmission electron microscopy (TEM), sometimes used together with cytochemical detection or biochemical measurement of lysosomal enzyme activities, and classical histological staining methods for light microscopy.

The first report with properly interpreted ultrastructural images of autophagic structures dates back to 1959 by Novikoff [6]. In the epithelial cells of proximal convolutions of kidneys in experimental hydronephrosis (caused by ligation of the ureter), mitochondria could be found in dense bodies that were positive for acidic phosphatase, a typical lysosomal enzyme [6, 7]. In 1962, Ashford and Porter published ultrastructural images of vesicles observed in hepatic cells of rats treated with glucagon, which obviously contained cytoplasmic material in various stages of degradation [8]. Subsequently, work in the laboratory of Christian de Duve, the biochemist famous for identifying and naming lysosomes, revealed that glucagon induced the relocalization of lysosomes to mediate glucagon-induced autophagy in rat liver [9]. Pfeifer published complementary studies on suppression of liver autophagy by insulin [10, 11]. Furthermore, starvation was already reported to be a strong enhancer of autophagy in rat liver back in 1964 [12]. It was de Duve who recommended to refer to the process of progressive degeneration of mitochondria and other organelles in cytolysosomes as autophagy (literally meaning “self-eating” in Greek), at a scientific meeting held in 1963 [13], and later described it in a widely cited review article [14]. It is worth noting that he also coined the names for processes now known as endocytosis (or heterophagy, which means “different eating” in Greek) and exocytosis in his lecture. A variety of terms were used initially for vesicles involved in autophagy, including initial and degrading autophagic vacuoles; these structures are now usually referred to as autophagosomes and autolysosomes, respectively.

Many of the pioneering early studies were carried out on insects other than* Drosophila*, as the fruit fly was not as popular before the revolution of molecular genetics as it is today. It was already shown in 1899 that in certain insects, the larval fat body (an organ with metabolic and storage functions similar to our liver and fat tissues) contains storage granules of proteins [15], and it was later described that honey bee larvae accumulated such granules just prior to pupation [16, 17]. The first recognition of autophagy in* Drosophila* melanogaster was published in 1963, showing TEM images of large autolysosomes containing ER and mitochondria in fat body cells of larvae approaching the time of puparium formation [18]. This programmed wave of autophagy in the larval fat body of holometabolous insects (those undergoing complete metamorphosis) is now known as an example of developmental autophagy.

In 1965, Locke and Collins provided a very detailed ultrastructural description of this process in the larva of the butterfly* Calpodes ethlius* [2]. Similar to the above examples, a large number of granules (which are essentially vesicles with a high protein content) form prior to metamorphosis in these animals. Three types could be distinguished: granules composed almost entirely of densely packed proteins that often form crystals, granules containing isolated regions of ER and mitochondria, and granules of a mixed type. This pioneering study published ultrastructural images that beautifully demonstrate phagophores in the process of capturing cytoplasmic contents such as a mitochondrion, double-membrane autophagosomes containing ER and mitochondria, and autolysosomes within which organelles are seen in various stages of degradation. Moreover, the authors properly recognized that the outer membrane of autophagosomes is involved in fusion with lysosomes (or first with each other), and after loss of the internal membrane, ER and mitochondria coalesce due to degradation by lysosomal enzymes. It is important to emphasize that the densely packed protein granules generated during this period originate in large part from the endocytic uptake of blood proteins when such holometabolous insect larvae (including* Drosophila*) are preparing for metamorphosis and that the heterophagy and autophagy pathways converge at the level of lysosomes [19–21]. It became clear that increases in the steroid hormone ecdysone trigger larval molts in these insects at a high concentration of juvenile hormone, and the drop in juvenile hormone concentration allows for the larval-pupal molt [22]. Note that in flies including* Drosophila*, first the larval cuticle hardens during puparium formation, and the actual molt only happens 5-6 h later, when the adult appendages such as legs and wings are everted from their primordia found as imaginal disks within the larval body. As early as in 1969, ligation and decapitation experiments (separating the ecdysone-producing endocrine organ from the larval fat body) were shown to prevent storage granule formation in* Calpodes*, and this effect could be rescued by injection of ecdysone [23]. In this report, Janet Collins already correctly hypothesized that ecdysone triggers autophagy only when juvenile hormone concentration is low, which was later confirmed in other insects including* Drosophila* [21, 24, 25].

Autolysosomes were also observed in ultrastructural images of* Rhodnius* larval fat body cells during prolonged starvation, published in 1967 by Wigglesworth [26]. Two years earlier, Francis Butterworth and colleagues reported that a 3-day starvation of early third instar* Drosophila* larvae induced massive granule formation in the fat body based on light microscopy [27], although this effect may have been due to the fact that once larvae reach the so-called 72 h checkpoint counted from the time of egg laying, they are able to initiate metamorphosis (and thus turn on developmental autophagy and heterophagy in the fat body) following acute starvation [28].

These early studies were not limited to the insect fat body. An ultrastructural analysis of eye development of wild-type and eye color mutants of* Drosophila* was published in 1966, demonstrating that the so-called type IV granules form in the pigment cells of various colorless mutants [29]. These granules are essentially autolysosomes as they were found to be positive for acid phosphatase and contained ribosomes, myelin-like membranes, glycogen, and ferritin [29]. In 1965, Lockshin and Williams showed that during the elimination of intersegmental muscles following adult ecdysis in silkmoths, increased activity of lysosomal cathepsins and acid phosphatases can be detected biochemically, and lysosome-like organelles abound which were later found to contain mitochondria [30–32]. These findings led to the morphological classification of this histolysis as a type II (or autophagic) cell death, to distinguish it from type I cell death events, which are characterized by the classical apoptotic morphology such as chromatin condensation, cell shrinkage, and blebbing [33].

## 3. Genetic Control of Autophagy in* Drosophila*

**Figure 1:**
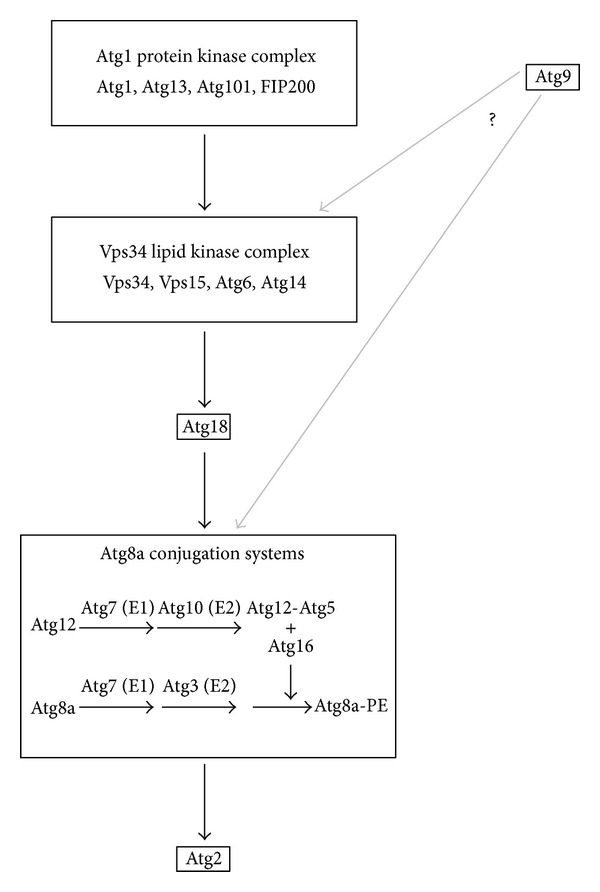
A model for the hierarchical relationships of Atg proteins in* Drosophila*. PE: phosphatidyl-ethanolamine. See text for details.

Multiple genetic screens carried out in the 1990's identified a core set of about 20 evolutionarily conserved genes required for autophagy in yeast [34–36]. Since different names were proposed often for the same genes in each screen, a consensus nomenclature for these* Atg* (autophagy-related) genes was adopted in late 2003 [37]. Note that the first study to demonstrate that an* Atg* gene homolog is also required for autophagy in a complete animal was published in* Drosophila* earlier that year, that is why it did not follow the agreed-upon naming conventions and referred to the fly homolog of* Atg3* as* Drosophila Aut1* [38]. It is commonly accepted that Atg gene products assemble into functional protein complexes, and several attempts have been made to establish their hierarchy during autophagosome formation in various models [39–41]. Such genetic epistasis analyses have proven difficult based on data from yeast and cultured mammalian cells, which is likely explained by the emerging connections between Atg proteins that were originally grouped into separate complexes, by temporal differences in the recruitment of various Atg proteins to phagophore assembly sites (PAS), and by differences in the localization of proteins thought to act as part of the same complex [4, 42, 43]. Nevertheless, we will discuss the role of these proteins according to the canonical classification in this review for clarity (please see also Figure 1).

The Atg1 complex is usually considered to act most upstream in the hierarchy of Atg gene products in all eukaryotic cells and contains the serine/threonine kinase Atg1 (the homolog of mammalian ULK1 and ULK2 proteins), Atg13, Atg101, and FIP200 (also known as RB1CC1 in mammals and Atg17 in flies) in metazoans. Of these, neither Atg101 nor FIP200 has clear homologs in yeast based on sequence comparisons, although FIP200 is thought to act similar to the scaffold protein Atg17 [44]. Biochemical studies in flies and mammals show that Atg13 directly binds to the other three subunits, and that it undergoes Atg1-mediated hyperphosphorylation upon starvation in* Drosophila* [44–46]. The catalytic activity of Atg1 seems to be especially important for autophagy induction. First, expression of kinase dead Atg1 inhibits autophagy in a dominant-negative fashion [47]. Second, overexpression of Atg1 strongly induces autophagy, which eventually culminates in cell death due to activation of caspases [47]. Third, Atg1 undergoes limited autophosphorylation during starvation, which is thought to increase its activity [44]. Interestingly, expression of dominant-negative, kinase dead Atg1 still shows a low-level rescue of the lethality of Atg1 null mutants [47]. Moreover, Atg1 was found to localize to the whole phagophore in yeast while all other subunits of this complex remain restricted to the initially appearing PAS area, indicating that Atg1 may also function independent of its canonical binding partners [43].

Both autophagosome and endosome membranes are positive for phosphatidylinositol 3-phosphate (PI3P), a phospholipid generated by the action of similar lipid kinase complexes. The core complex contains Atg6 (known as Beclin-1 in mammals), the catalytically active class III phosphatidylinositol 3-kinase (PI3K) Vps34, and its regulatory subunit Vps15, which has a serine/threonine kinase domain. A catalytically inactive point mutant of Vps15 was shown to lose Vps34 binding in yeast [48], but the significance of its putative protein kinase activity is poorly understood. The identity of the fourth subunit is critical: Atg14 is present in the autophagy-specific complex while the other complex involved in endocytosis contains UVRAG/Vps38, and the binding of these subunits to the core complex has been shown to be mutually exclusive in mammalian cells [49, 50]. Starvation-induced autophagy is severely impaired in Vps34 null mutant or dominant-negative Vps34 overexpressing cells, although some autophagosomes form at a reduced rate [51]. This may be explained by the activity of the class II PI3K, which was suggested to partially compensate for the loss of Vps34 during autophagy in mammalian cells [52, 53]. Similarly, deletion of* Drosophila* Vps15 or Atg6 results in a block of starvation-induced autophagy [54, 55]. In line with the distinct roles of different Vps34 complexes in mammals and yeast, it has been shown that* Drosophila* UVRAG is involved in endolysosome maturation and is dispensable for autophagosome formation or fusion with lysosomes, whereas studies using RNAi or hypomorphic mutants suggested that Atg14 is required for autophagy in larval fat body cells [56–59].

It is commonly accepted that PI3P found on phagophore and autophagosomal membranes recruits and activates phospholipid effectors. One class of such proteins includes the metazoan homologs of the yeast WD40 domain protein Atg18, which are called WIPI1-4 in mammals [60, 61]. In* Drosophila*, Atg18 has been shown to be required for autophagy, whereas the function of its closely related paralog CG8678 (also known as Atg18b) is not known [62]. DFCP1 (double FYVE containing protein 1) was characterized as another phospholipid effector, and it translocates to a putative subdomain of the ER during autophagy induction [63]. This structure is called the omegasome, and it is also positive for VMP1 (vacuole membrane protein 1), an ER-localized, six transmembrane domain containing protein of poorly characterized function [40, 64]. Interestingly, VMP1 has been found to interact with Beclin-1, suggesting that it may modulate phospholipid production [65]. The fly homolog of VMP1 is called Tango5 (Transport and Golgi organization 5), as it was recovered in a cell culture-based RNAi screen as required for ER to Golgi trafficking in the secretory pathway [66]. Interestingly, the gene encoding DFCP1 has been lost multiple times during evolution as it is missing from all* Caenorhabditis* and most* Drosophila* species including* Drosophila melanogaster*, but its homolog can be clearly identified in* Drosophila willistoni *and the* virilis* subgroup using bioinformatic searches, in addition to more ancient species such as* Trichoplax* and* Hydra*. The role of DFCP1 is also unknown in mammals, and it is mostly used as a marker along with VMP1 for the PAS [40, 42].

Atg9 is the only transmembrane protein among the Atg gene products identified in yeast, and it likely plays a critical role in the membrane transport events during phagophore assembly in all eukaryotes studied so far [42, 67–69]. The source of autophagic membranes has been debated since the discovery of this process, and practically all membrane compartments were suggested to contribute, including endosomes, ER, Golgi, mitochondria, and plasma membrane [70–72].* Drosophila* Atg9 is still largely uncharacterized, with only a few RNAi studies showing that it is also required for autophagy in various settings [57, 73–75]. Yeast Atg9 physically binds to Atg18 and Atg2, and these proteins are required for the retrograde traffic of Atg9 from the PAS in yeast [76]. Atg9 also binds to fly Atg18, and it has recently been shown that Atg9 accumulates on protein aggregates containing the autophagy cargo Ref(2)P (also known as p62/SQSTM1) in starved Atg7, Atg8a, and Atg2 mutants, but not in Atg18 mutants [75].

**Figure 2:**
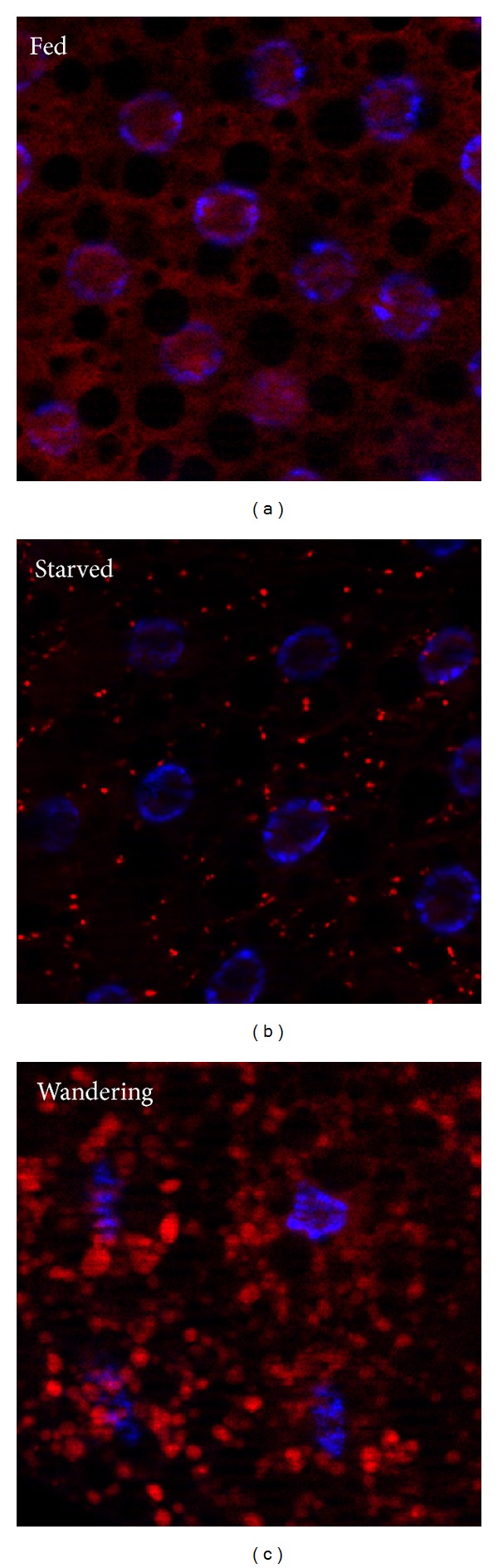
Autophagy induction in the larval* Drosophila* fat body. Dots positive for mCherry-Atg8a (red), representing autophagosomes and autolysosomes, are rarely seen in fat body cells of well-fed larvae (a). Punctate mCherry-Atg8a structures form in response to starvation (b) or during the wandering period (c). DNA is stained blue.

Structural studies of Atg8 and Atg12 revealed that these proteins belong to the family of ubiquitin-like modifiers, and these are involved in two related ubiquitin-like conjugation systems [77]. First, the C-terminal amino acid(s) following a glycine residue of Atg8 and its homologs are cleaved by the Atg4 family of cystein proteases. Subsequently, the exposed glycine is conjugated to the E1-like enzyme Atg7, followed by its transfer to the E2-like Atg3 (also known as Aut1 in flies). In parallel, Atg12 is activated by Atg7 as well, and then the E2-like Atg10 catalyzes the formation of an Atg5-Atg12 conjugate [77]. Atg5 contains two ubiquitin-related domains flanking a helical region [78]. Then, a multimeric complex of Atg5-Atg12 and Atg16 forms, which enhances the covalent conjugation of Atg8 to the membrane lipid phosphatidylethanolamine (PE) [78]. Atg8 and its homologs (Atg8a and Atg8b in flies, and LC3 and GABARAP family proteins in mammals) are the most commonly used markers in autophagy studies [40, 79]. First, Atg8 is covalently bound to phagophore and autophagosome membranes, making it possible to visualize these structures using tagged reporters or by immunostaining using antibodies against endogenous proteins (Figure 2). Second, the processing of Atg8 can be followed by Western blots, as unconjugated Atg8 (usually referred to as Atg8-I or LC3-I) migrates slower than the lipid-bound form (Atg8-II or LC3-II). Autophagy induction usually increases the amount of the processed form relative to tubulin or actin, which becomes even more obvious if the fusion of autophagosomes with lysosomes is blocked by bafilomycin, or genetically by loss of the autophagosomal SNARE Syntaxin 17 [79–82].

It is clearly established that Atg2 and Atg18 function together in yeast, acting most likely in parallel to the Atg8 and Atg12 conjugation systems [39, 83]. In mammals, depletion of the Atg18 homolog WIPI2 suppressed LC3 puncta formation [61]. In contrast, its putative binding partner Atg2 appears to function most downstream of the core Atg genes in mammals and worms, similar to VMP1 homologs, as Atg8-positive structures with some characteristics of phagophores form in cells upon silencing of these genes [40, 41, 64, 84]. Atg18 also shows an interaction with Atg2 in* Drosophila*, although it is weaker than that observed between its paralog CG8678 and Atg2 [75]. Interestingly,* Drosophila* Atg2 acts downstream of, or parallel to, the Atg8 systems in* Drosophila* as well, as it is dispensable for Atg8a dot formation in the fat body [75, 80]. In contrast, no GFP-Atg8a puncta were seen in Atg2 mutant prepupal midguts [85], suggesting that either tissue-specific differences exist, or that a GFP-Atg8a reporter expressed at very low levels is not as potent as anti-Atg8a immunolabeling for the visualization of these aberrant structures that are apparently seen in most metazoan cells. This issue clearly warrants further studies.

*Drosophila* Atg18 appears to function upstream of Atg8 recruitment during phagophore formation similar to worms and mammals, as punctate Atg8a localization is lost in Atg18 mutant or RNAi cells [41, 61, 75, 84]. Interestingly, protein aggregates positive for ubiquitin and Ref(2)P show a near complete colocalization with FIP200 and Atg9 in* Drosophila* mutants lacking more downstream players, raising the possibility that such protein aggregates may serve as an organizing centre during autophagosome formation [46, 75]. This hypothesis will need further testing.

A complicated network of core Atg proteins coordinates the process of autophagosome formation, a process that is still not completely understood. Autophagosomes must fuse with lysosomes and endosomes to deliver their cargo for degradation. In yeast, direct fusion of the autophagosome with the vacuole is achieved by a tethering factor called HOPS (homotypic fusion and vacuole protein sorting) complex, which facilitates membrane fusion catalyzed by SNARE proteins Vam3, Vam7, and Vti1 [86]. Interestingly, autophagosome fusion in* Drosophila* appears to depend on the amphisome pathway, as a genetic block of multivesicular endosome formation results in large-scale accumulation of autophagosomes [51, 87]. Recent studies identified Syntaxin 17 as the autophagosomal SNARE protein, both in flies and mammals [80, 81]. Syntaxin 17 binds to ubisnap, an ortholog of mammalian SNAP-29, to mediate fusion by forming a ternary complex with late endosomal/lysosomal VAMP7 (VAMP8 in mammals) [80, 81]. Fusion is facilitated by the binding of HOPS to this SNARE complex, both in* Drosophila* and mammalian cells [58, 88]. In the final steps following fusion, cargo is degraded inside acidic autolysosomes by the action of hydrolases such as cathepsins, and the breakdown products are recycled back to the cytosol to fuel synthetic and energy producing pathways.

## 4. Regulation of Autophagy during* Drosophila* Development

The best known examples for stimulus-induced autophagy in* Drosophila* larvae are the starvation response during the feeding stages and developmental autophagy triggered by hormonal cues around the start of metamorphosis in polyploid tissues. The role and regulation of autophagy have also been studied in a developmental context in adult ovaries and in the extraembryonic tissue called amnioserosa during early embryogenesis. The following paragraphs summarize the major regulatory pathways regulating autophagy in these settings.

Autophagy is controlled by the main nutrient and energy sensor in all eukaryotic cells, a serine/threonine kinase called TOR (target of rapamycin) [89]. TOR activity is increased by the presence of nutrients and growth factors and promotes cell growth in part through the phosphorylation and activation of S6k (RPS6-p70-protein kinase) and phosphorylation and inactivation of Thor (also known as 4E-BP for Eukaryotic translation initiation factor 4E binding) [90]. TOR not only enhances general protein synthesis this way, but it may also increase net cell growth by actively repressing autophagy through the direct phosphorylation and inhibition of Atg1 in metazoans [45, 91–93]. Inactivation of TOR during starvation, growth factor withdrawal, or impaired lysosomal function rapidly results in the shutdown of cap-dependent translation and in the activation of autophagy, which is likely also facilitated by the poorly characterized action of phosphatases such as PP2A that may antagonize TOR [52, 56, 62, 91–94]. Interestingly, the serine/threonine kinase Atg1 and its mammalian homologs are able to directly phosphorylate TOR, which may act as a feedback mechanism to inhibit cell growth and further enhance autophagy induction [47, 95]. Growth signaling pathways are remarkably active in the larva, a specialized life stage of holometabolous insects. Larvae basically just eat and grow throughout the feeding stages to acquire and store as many nutrients as possible in a relatively short time, mostly in the form of polyploid cells and tissues besides the hemolymph. Notably, the size of the larval fat body (a metabolic organ similar to our liver and white fat tissues) increases more than 200-fold between the first and mid-third instar stages in* Drosophila*. This process generates polyploid cells of enormous size, reaching a ploidy level of 256–512 n for fat cells and 1,024 n for salivary glands. As expected, autophagic activity is very low during these stages (Figure 2). Initiation of wandering behavior, when larvae crawl out of the food in search of a dry place to pupariate around 108 h after egg laying (AEL), or starvation before this time results in a remarkable induction of autophagy in polyploid tissues (Figure 2), but not in diploid cells. This response is thought to serve as a nutrient reallocation mechanism, as breakdown products released from polyploid cells likely feed diploid tissues that will give rise to the adult fly by the end of metamorphosis. Mechanistically, growth signaling mediated by the insulin-like receptor is rapidly inactivated during starvation or at the beginning of metamorphosis in polyploid tissues [62, 96]. Diploid tissues such as the brain and wing disc appear to be able to grow and proliferate thanks to maintained activation of TOR signaling by sustained receptor Tyrosine kinase signaling, originating from Alk in neurons and Stit in future wing cells, respectively [97, 98]. In addition, the larval fat body secretes an insulin-like peptide (dilp6) during nonfeeding stages to maintain insulin signaling in diploid tissues [99].

As described briefly in the chapter on historical early studies, autophagy of the polyploid tissues including fat body and midgut cells is induced by a small peak of the molting hormone ecdysone towards the end of the last larval instar [20, 96]. Interestingly, there is a preprogrammed anterior-posterior gradient in the magnitude of autophagy in the fat body [100]. This is also observed for the separation of fat cells and kynurenine synthesis during metamorphosis, potentially due to the extremely low blood circulation in sessile prepupae and pupae, which necessitates the coordination of all these responses with respect to the location of nearby imaginal organs [100, 101]. Autophagy is induced in fat body cells as a cell-autonomous response, as overexpression of dominant-negative forms of the ecdysone receptor in mosaic animals maintains insulin signaling and blocks developmental autophagy in these cells [96]. Massive induction of autophagy is not seen during earlier ecdysone peaks that trigger larval molts, because high concentration of the juvenile hormone during the first and second larval stages inhibits autophagy. It is not known yet how juvenile hormone may inhibit autophagy. One candidate mechanism involves the peptidyl-prolyl cis-trans isomerase FKBP39. FKBP39 is a juvenile hormone target gene, and it has been shown to inhibit autophagy likely by preventing the translocation of the transcription factor FOXO into the nucleus [102, 103]. The presence of FOXO in the nucleus during starvation or at the beginning of metamorphosis likely promotes transcription of genes involved in autophagy, and its loss strongly impairs autophagic responses [103, 104]. It is worth mentioning that metamorphosis is not the only developmentally programmed starvation period in* Drosophila*, as larvae are also essentially immobile and do not feed during periods of molting that separate L1/L2 and L2/L3 stages, leading to increased autophagy in fat body (Gábor Juhász, unpublished data). This response is similar to the induction of autophagy observed during molting in worms [105].

Polyploid cells that account for the majority of larval masses undergo programmed cell death during metamorphosis. Initially, the larval fat body disintegrates into individual trophocytes following puparium formation, which is triggered by a prominent ecdysone peak at the end of the last larval instar [106]. Interestingly, approximately half of the larval fat cells survive until eclosion of adult flies and are only eliminated by caspase-dependent cell death during the first two days of adult life, promoting the survival of starved young adults [107, 108]. Salivary glands are also almost entirely composed of polyploid cells in the larva, with the exception of a ring of diploid imaginal cells surrounding the ducts of the paired glands. Larval gland cells are eliminated around 13–18 h after puparium formation, and both autophagy and activation of apoptotic caspases have been shown to facilitate histolysis, although the relative importance of each pathway is not fully understood [109–114]. A wave of autophagy is also seen in larval midgut cells of wandering larvae, but their elimination begins only after puparium formation, and it is not completed until after adult flies eclose [96, 115]. Groups of diploid imaginal cells (scattered throughout the larval gut) proliferate and replace polyploid cells during this process. Thus, polyploid cells are extruded into the lumen of the future adult gut, which is accompanied by caspase activation, DNA fragmentation, and autophagy-mediated shrinkage of these larval cells [85, 110, 112, 113, 115]. Remnants of the larval midgut form the meconium, the waste product that adult flies get rid of during the first defecation.

There is some discrepancy regarding the role of the apoptotic and autophagic pathways during larval* Drosophila* midgut degeneration. Two papers suggested that midgut shrinkage is blocked by expression of the caspase inhibitor p35, or by simultaneous loss of two proapoptotic genes Rpr and Hid [110, 112]. Importantly, RNAi depletion of the caspase inhibitor DIAP1 leads to premature caspase activation and death of larval midguts and salivary glands [110]. In contrast, midgut shrinkage was suggested to proceed largely independent of caspase activation based on experiments carried out on animals with a combination of mutations for certain caspases, whereas midgut cells fail to shrink properly if certain* Atg* genes are silenced or mutated [85, 115]. Interestingly, overexpression of Hid in* Drosophila* larvae triggers apoptosis in diploid cells of the developing eye and brain, but it leads to the induction of autophagy in polyploid cells of the fat body, salivary glands, and midguts [116], also indicating tissue-specific differences in the mechanism of action of certain proapoptotic genes.

In contrast to ecdysone-mediated shutdown of insulin signaling, which is responsible for the initial wave of autophagy in wandering animals, death of polyploid cells in salivary glands and midguts appears to be regulated by a complex transcriptional cascade. As mentioned earlier, the elimination of about half of the fat body cells takes place in the pupa in a seemingly random manner, and surviving cells only die in young adults [108]. In prepupal midguts and pupal salivary glands, binding of ecdysone (or more likely its active form 20-hydroxyecdysone) activates the heterodimeric steroid receptor complex consisting of EcR and USP (the homolog of mammalian retinoid X receptor). Activation of this complex by ecdysone is necessary to trigger salivary gland cell death by inducing transcription of insect-specific target genes such as E93, E74A, and BR-C, but this process also requires a competence factor: the nuclear receptor *β*FTZ-F1 [117]. E93 is a transcription factor acting as a master regulator of the complex genetic programme involved in the death of both larval salivary glands and midgut in* Drosophila* [114, 118]. The role of autophagy in dying salivary gland and midgut cells may not be restricted to the recycling of building blocks to support diploid cells. Autophagy in dying mammalian cells is known to promote the release of so-called “eat me” and “come get me” signals to attract engulfing macrophages [119]. While larval midgut cells are situated inside the adult gut and are therefore protected from hemocytes, clearance of salivary gland cell fragments may be facilitated by macrophages in the pupa. This hypothetical scenario would explain why salivary glands undergo complete histolysis, whereas midgut cell remnants remain in the lumen of the adult gut until excreted.

Given the seemingly important role of autophagy during* Drosophila* development, it is surprising that null mutants for different genes show large differences regarding viability. Null mutants of* Atg1*,* Atg13*, and* FIP200* display a highly penetrant pharate adult lethality: adult flies form completely inside the pupal case, but almost all of them fail to eclose [45–47, 120]. The lipid kinase complex subunit null mutants (*Atg6, Vps34*, and* Vps15*) die much earlier (as L3 stage larvae), and only a few* Atg6* mutants are able to initiate pupariation [51, 54, 55]. This is not surprising considering that these gene products are involved in endosome maturation and biosynthetic transport to lysosomes acting in a complex with UVRAG. It is worth noting that UVRAG null mutants also die as late L3 stage larvae, even though UVRAG is dispensable for autophagosome formation or fusion with lysosomes [58, 121]. It will be interesting to see the phenotype of flies null mutant for* Atg14*, which encodes the autophagy-specific subunit of this complex, as these should behave similar to Atg1 kinase complex subunits in showing pharate adult lethality. Similarly, both* Atg2* and* Atg18* mutants are late pupal/pharate adult lethal. In contrast, all null mutants identified so far in genes encoding proteins involved in the ubiquitin-like conjugation systems are viable, including* Atg7* [113],* Atg8a* [57, 122], and* Atg16* (Gábor Juhász, unpublished data). Moreover, these null mutants can be maintained as viable stocks over multiple generations despite their shorter lifespan and increased stress sensitivity. The reason why null mutations affecting conjugation system components are viable in* Drosophila* is not known. A recent paper showed that prepupal midgut shrinkage requires Atg8a and Atg16, but not Atg3 or Atg7 [115], suggesting that Atg8a promotes cell shrinkage in a lipidation-independent manner. Still, these results do not explain the lethality data described above. Potential explanations can be that certain* Atg* genes are not required for autophagy in certain key developmental settings (such as* Atg3* and* Atg7* in midgut shrinkage), or that the ones that are lethal also have important roles independent of autophagic degradation (similar to* Vps34*,* Vps15,* and* Atg6*). It is important to note that* Atg3, Atg5, Atg7, Atg9,* and* Atg16L1* knockout mice complete embryonic development and are born at expected Mendelian ratios and only die due to suckling defects, whereas the loss of beclin* 1/Atg6* leads to lethality during early embryogenesis [4].

Another role of autophagy has been described in the* Drosophila* ovary. During oogenesis, 15 nurse cells transfer a large part of their cytoplasm to the single oocyte through interconnecting cytoplasmic bridges called ring canals. Nurse cells die after the oocyte has matured, which is accompanied by caspase activation and DNA fragmentation. Caspase activation is reduced in nurse cells lacking Atg1, Atg13, or Vps34, and both DNA fragmentation and cell elimination are reduced [123]. Interestingly, the antiapoptotic protein Bruce accumulates in these mutant cells. Bruce colocalizes with GFP-Atg8a in wild-type ovaries, and loss of Bruce restores nurse cell death in autophagy mutants [123]. These observations suggest that autophagic elimination of Bruce may contribute to caspase activation and cell death in late stage* Drosophila* ovaries. However, mutation of either core autophagy genes or caspases, or the simultaneous loss of both autophagy and caspases still results in only a partial inhibition of developmental nurse cell death [124]. In contrast, hypomorphic mutation of dor/Vps18, a subunit of the HOPS complex, blocks nurse cell elimination much more efficiently, suggesting that lysosomes or endocytosis may play a more important role in developmental nurse cell death than autophagy or caspases [124, 125].

Autophagy can also be induced in the ovary during two earlier nutrient status checkpoints in germarium and mid-oogenesis stages, both in nurse cells and follicle cells, somatic epithelium surrounding germ cells [126–128]. This autophagic response requires core Atg genes and the caspase Dcp-1, and it can be suppressed by overexpression of Bruce [126, 127]. Interestingly, oogenesis is impaired in chimeric ovaries lacking autophagy in a subset of follicle cells but not in the germline, which may be caused at least in part by precocious activation of Notch signaling in mutant follicle cells [127, 129].

Another example for developmentally programmed autophagy is seen in the amnioserosa, a polyploid extraembryonic tissue of the developing embryo. Autophagy is induced prior to, and independent of, the activation of a caspase-dependent cell death programme in these cells [130]. Autophagy is also activated in a subset of amnioserosa cells that undergo extrusion during dorsal closure, but it is not required for the death of these cells [131].

In contrast with the paradigm of the inverse regulation of cell growth and autophagy by TOR signaling, autophagy has been shown to be required for cellular overgrowth driven by the evolutionarily conserved transcription factor Myc. Myc is required for autophagy, both in* Drosophila* and mammalian cells [73, 132]. Conversely, overexpression of this well-known oncogene not only enhances cell growth, but it also leads to autophagy induction through activation of PERK, an ER-associated kinase involved in the unfolded protein response (UPR). Importantly, blocking PERK or autophagy prevents Myc-induced overgrowth in* Drosophila* and inhibits Myc-induced tumorigenesis in mouse models [73, 133]. These results suggest that inhibition of PERK or autophagy may be a potential therapeutic strategy in the context of Myc-dependent cancers.

## 5. Autophagy Implication in the Immune Response, Aging, and Neurodegeneration

Autophagy plays an important role in development, cellular differentiation, and homeostasis. Defects in autophagy are associated with many diseases including neurodegeneration, ageing, pathogenic infection, and cancer [5].* Drosophila* melanogaster has been shown to be an excellent model system to study such cellular processes. The key advantages of using* Drosophila* as a disease model organism are short life cycle, small body size, ability to produce large number of progeny, availability of powerful genetic tools, and less redundant genome than that of mammals. Moreover, more than 70% of human disease genes have orthologues in* Drosophila* [134].

Autophagy has also been proposed to play a role in the removal of pathogens, given that it is the only degradative system in the cell which is able to handle cargo that is too large for proteasomal degradation. Evidence shows that autophagy is able to capture and degrade multiple categories of pathogens, including bacteria, viruses, and parasites [135]. This is not, however, a universally effective defence system, as some pathogens have developed resistance against it, or even learnt how to use autophagy in order to enhance their own replication [135, 136]. This interplay between host defences and infective agents suggests that autophagy, as an intracellular immune response, has exerted strong selective pressure on pathogens over the course of a long evolutionary time [137]. Flies lack an adaptive immune system, which facilitates the study of autophagy-derived innate immunity at the cellular level, without added complexity [138].

*Drosophila* has also been used successfully to study of the effects of pharmacological modulators of autophagy in neurodegenerative disease models. The available* Drosophila* disease models successfully recapitulate many of the symptoms associated with human diseases, and these can be used to identify new factors with a role in diseases [134].

### 5.1. Autophagy-Derived Innate Immunity

In mammals, pathogen recognition activates the antimicrobial response of the host, using transcription level regulators [137]. So far, two well-characterised nuclear factor-*κ*B (NF-*κ*B) pathways are known in flies: the Toll and immune deficiency (IMD) pathways, which are key to regulating the immune response against bacterial and fungal infections, by means such as the secretion of antimicrobial peptides (AMPs) [138, 139]. The Jak-Stat pathway, native to higher organisms, also plays a role in the immune defence response in flies, and all of the aforementioned pathways have been observed to mediate antiviral responses at the level of transcription [140, 141]. There are many aspects of the innate immune response in insects which are yet to be elucidated, and the role of autophagy in the antimicrobial response is only beginning to be deciphered. Striking parallels were observed between flies and mammals in terms of antimicrobial functions of autophagy [137]. A new aspect in mammalian antimicrobial autophagy, which is quickly gaining visibility, is the role of pattern recognition receptors (PRRs) in the activation of autophagy [135, 142]. These receptors work by recognising well-conserved molecular signature sequences, called pathogen-associated molecular patterns (PAMPs) [143]. The* Drosophila* protein Toll was first used to pinpoint the mammalian Toll-like receptors (TLRs) by virtue of homology, which make up the canonical pattern recognition system [137, 138]. These membrane receptors can induce autophagy upon binding to a cognate ligand [144]. Their cytoplasmic counterparts, the NOD-like receptors (NLRs), can activate autophagy as well [145, 146]. The importance of autophagy control by PRRs in mammalian host defence is certainly an interesting research avenue, despite the difficulty of assessing its* in vivo* potential during infection in mice.* Drosophila*, on the other hand, offers a much more genetically malleable system for such studies. The relationship between autophagy and PRRs has been found to be critical in preventing the host from succumbing to viral and bacterial infections [137]. Hence, it is likely that antimicrobial autophagy is an ancient cellular response to invading pathogens.

Autophagy genes have been shown to confer resistance to parasites (*Toxoplasma gondii*), bacteria (*Staphylococcus aureus*,* Listeria monocytogenes*,* Salmonella enterica*,* Typhimurium,* and* Mycobacterium tuberculosis*), and viruses (Sindbis virus, vesicular stomatitis virus (VSV), and herpes simplex type 1) [147–154]. Importantly, a landmark study recently showed that parkin, a gene implicated in the pathogenesis of Parkinson disease by promoting the selective autophagic elimination of mitochondria, is also important for the recognition and subsequent autophagic degradation of infecting intracellular bacteria in mice and* Drosophila* [155].

In terms of bacterial resistance, the* Drosophila* immunity comes equipped with two previously mentioned major response pathways: the Toll pathway, which is usually activated by Gram-positive bacteria, and the IMD pathway, which mainly handles Gram-negative bacteria [138]. Activation of either of these systems depends on the receptors' ability to detect PAMPs, such as the bacterial cell wall component peptidoglycan (PGN) [138]. This process and the subsequent release of AMPs are vital given that flies that are deficient in either the IMD or Toll pathway display hypersusceptibility to bacterial infection [156].

There are, however, species that show resistance to such a host response. Both the IMD and Toll signalling pathways are dispensable for controlling intracellular* L. monocytogenes* in flies. Instead, once bacteria have escaped to the cytoplasm, autophagy restricts their replication.* L. monocytogenes* replication takes place in the cytoplasm of* Drosophila* blood cells, termed “haemocytes” [157]. It has been observed that* L. monocytogenes* induces autophagy, which was visualised by the appearance of GFP-fused LC3 puncta that colocalised with internalised bacteria [157]. This study showed that RNAi-mediated silencing of core autophagy genes causes increased bacterial replication and reduces fly life expectancy in infected adults.

In mammalian cells, autophagy can also degrade* L. monocytogenes*, but this process is normally blocked by the release of ActA, which inhibits the host's ability to ubiquitinate the pathogen and target it for autophagosomal degradation [153]. A similar autophagy evading behaviour has been independently observed in conjunction with protein InlK, although the mechanism is yet unexplained [158]. Failure to successfully resist the host's response, such as in the unnatural host* Drosophila*, reveals restrictive pathways that the* L. monocytogenes* cannot evade and highlights the constant adaptations that the bacterium must undergo in order to effectively counteract the immune responses of the host [137]. Upstream of the IMD pathway is the PGN recognition protein (PGRP) family receptors, which recognize bacterial PGN structures. PGRP-LC is a transmembrane sensor, which recognises monomeric and polymeric diaminopimelic acid- (DAP-) type PGN at the cell surface. PGRP-LE comes in two forms that have both cell-autonomous and non-cell-autonomous functions [159]. It is constitutively secreted into the open circulatory system, where it activates the IMD pathway [160]; it is also found within immune cells and acts as an intracellular receptor for the detection of the PAMP tracheal cytotoxin, a monomeric DAP-type PGN, initiating the release of the listericin AMP [161, 162]. Loss of either of the two receptors confers susceptibility to infection by* L. monocytogenes*, but only PGRP-LE initiates autophagy as an immune response. Unexpectedly, PGRP-LE can signal via the IMD pathway, components of which are not required either for autophagy induction or intracellular bacterial sequestration, suggesting that an unknown signalling pathway links PRR engagement to antimicrobial autophagy in* Drosophila*. Autophagy is observed to play an important regulatory role against a variety of bacterial invaders. Multiple hosts have been found to utilise autophagy to control the growth of* Wolbachia*, a common endosymbiotic bacterium, found in arthropods and filarial nematodes. Activation of autophagy by starvation or rapamycin treatment was found to reduce the rate of bacterial replication; conversely, siRNA-mediated depletion of Atg1 in flies was associated with enhanced bacterial replication [163].

In addition to controlling bacterial infection, autophagy was found to impact viral replication and pathogenesis in some mammalian infections [137]. Overexpression of beclin-1 (mammalian homologue of Atg6) in neonatal mice protects neurons against Sindbis virus infection-induced pathogenesis [164]. Loss of Atg5 expression accelerates the development of Sindbis-associated symptoms, due to failed viral capsid clearance, even though autophagy does not appear to affect viral replication proper [150]. A range of other viral agents are ostensibly managed by autophagy, such as HIV, encephalomyocarditis virus, and human papilloma virus in mammalian cells, although the* in vivo* significance has not been weighed [165, 166].

**Figure 3:**
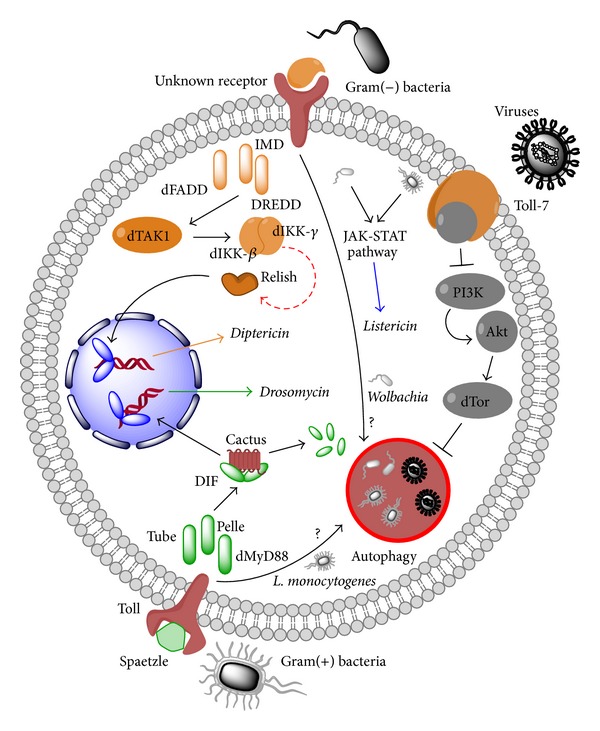
*Drosophila* immunity response pathways. A robust innate immunity system confers* Drosophila* protection against a variety of pathogens. Autophagy has been suggested to play a role in restricting infections, but the exact pathway of this response has yet to be deciphered. In addition there have been observations of a number of antimicrobial peptides (e.g., Diptericin) being expressed in response to immunological challenge.

Recent data demonstrates that autophagy is a key element of the innate antiviral response against (−) ssRNA Rhabdovirus VSV in flies [151]. Negative sense viral RNAs must be first converted into mRNA-like positive-sense strands by an RNA polymerase, before they can be translated. Depletion of core autophagic machinery genes in* Drosophila* S2 cells leads to increased viral replication. Along the same lines, RNAi silencing of autophagy genes was associated with increased viral replication and mortality after infection of flies, directly linking autophagy with an important antiviral role* in vivo* [151]. VSV was observed to induce PI3 K-Akt regulated autophagy in primary haemocytes and in adult flies [151]. Similar to the immune response against* L. monocytogenes* infection, antiviral protection is also initiated by the recognition of PAMPs [151]. An active response against UV-inactivated VSV suggested that nucleic acids are not the targeted markers; rather, the viral glycoprotein VSV-G was sufficient to induce autophagy. Eventually, the* Drosophila* Toll-7 receptor was identified as the PRR, which identifies VSV as a trigger for an autophagic response [167]. Toll-7 is localised to the plasma membrane in order to interact with the virions, suggesting that the roles of Toll-7 and the mammalian TLRs are similar. Toll-7 restricts VSV replication in cells as well as in adult flies, as deficiency of Toll-7 leads to significantly increased mortality after infection [167]. Recent work has drawn in other Toll receptors as likely participants in the host's immune response. Tollo (Toll-8) has been shown to negatively regulate AMP expression in* Drosophila* respiratory epithelium [168]. Many antiviral factors are upregulated during infection; given that* Drosophila* Toll and Toll-7 receptors have been recently shown to be transcriptionally induced upon infection, it is possible that the other less characterised Toll receptors may also play a role in antiviral defences (Figure 3).

There is an overlap in the mode of action of Toll receptors and mammalian TLRs in triggering autophagy. A number of studies using model ligands and in vitro systems have shown autophagy induction via the TLR pathway (such as lipopolysaccharide, a ligand for TLR4, by looking at the colocalisation of autophagosome markers and intracellular bacteria) [169]. Autophagic activation can be observed using canonical ligands for TLR1, TLR3, TLR5, TLR6, and TLR7 [144, 170]. TLR8 was revealed in a recent study to activate vitamin D-dependent autophagy in human macrophages, in order to restrict HIV replication [137, 171].

### 5.2. Autophagy in Ageing and Life Span Extension

Ageing is a complex process that involves a progressive decline in physiological functions of an organism, eventually causing disease and death [172]. During this decline, cellular and molecular damage accumulates such as deleterious mutations, shortening of telomeres, accumulation of ROS, damaged organelles, and misfolded proteins. Aged individuals have increased sensitivity to environmental stress and a decreased capacity to maintain cell and tissue homeostasis. Prevalence of many diseases such as neurodegeneration, cardiovascular dysfunction, and cancer increases with age [173].

Autophagy maintains cellular homeostasis by targeting unwanted and deleterious intracellular materials to the lysosome for degradation. Autophagy has been implicated in numerous diseases [5]. Accumulating evidence indicates that the efficiency of autophagy decreases with age, and the induction of autophagy delays aging-associated symptoms and extends life span [172]. In addition to the direct effect of autophagy on ageing, cellular pathways with a role in regulating ageing are shown to induce autophagy as their downstream targets [174–176]. These highly conserved pathways are insulin/insulin like growth factor (Igf) (ISS) pathway, the TOR pathway, c-Jun N-terminal kinase (JNK) signaling, and histone deacetylation [174, 177].

During ageing, the expression levels of several autophagy genes are downregulated in mammals. Autophagy mutants often exhibit phenotypes such as the accumulation of ubiquitinated protein aggregates, damaged organelles, increased sensitivity to oxidative stress, abnormal motor function, and short life span that are similar to those observed during ageing [172]. The expression level of Atg5, Atg7, and Beclin-1 is downregulated in human brains during ageing [178, 179]. Furthermore, a decrease in Beclin-1 expression has been reported in the brains of patients with Alzheimer's disease (AD) and Huntington's disease (HD) [179, 180]. Disruption of autophagy by reducing Beclin-1 expression enhances the severity of neurodegenerative phenotypes in transgenic APP (amyloid precursor protein) mice, and overexpression of Beclin-1 was sufficient to rescue the adverse effects in APP transgenic mice [180]. Suppression of basal autophagy in the central nervous system causes neurodegenerative phenotypes in mice even in the absence of a toxic protein: mice lacking Atg5 or Atg7 specifically in the central nervous system exhibit behavioural defects, motor dysfunction, accumulation of protein aggregates, and reduced life span [181, 182]. Chaperone-mediated autophagy (CMA) has been shown to be downregulated in rat livers during ageing as well. Restoring the level of chaperone-mediated autophagy by overexpressing LAMP2a, a CMA receptor, decreased the accumulation of damaged proteins and increased organ function [183]. A reduction in autophagy levels is also observed in mice during ageing. The heart-specific deletion of Atg5 causes abnormal heart morphology and the accumulation of abnormal protein aggregates and damaged mitochondria in mice [184].

Similar to these observations in mammals, the expression of several autophagy genes (Atg2, Atg8a, Atg18, and bchs) is reduced in* Drosophila* during ageing. This correlates with an increase in accumulation of insoluble ubiquitinated protein aggregates (IUP) in the ageing brain [122].* Drosophila* Atg8a mutants exhibit reduced autophagy, increased accumulation of IUP, increased sensitivity to oxidative stress, and reduced life span. Overexpression of Atg8a in adult brains decreased the incidence of IUP and increased oxidative stress tolerance and life span [122]. Similarly,* Drosophila* Atg7 null mutants are hypersensitive to nutrient and oxidative stress. Atg7 null mutants exhibit reduced life span and progressive neurodegeneration, which is characterized by the accumulation of ubiquitinated proteins [113]. Overexpression of Atg7 increases life span in wild-type flies and also rescues the age-related phenotypes caused by the knockdown of Hsp27 chaperone in* Drosophila*. Interestingly, overexpression of Hsp27 also extends life span in wild-type flies and rescues the neurodegenerative phenotypes caused by mild polyQ toxicity. The Hsp27-mediated rescue effect is abolished in flies lacking Atg7 [185]. Loss of the autophagosomal SNARE Syntaxin 17 has severe consequences: young mutant adults perform extremely poor in standard climbing tests that measure neuromuscular function and die within 3-4 days of eclosion. This is potentially due to large-scale accumulation of autophagosomes in neurons which causes neuronal dysfunction, rather than to cell death, as the lethality and behavior defects cannot be rescued by genetic inhibition of caspases in Syntaxin 17 mutant brains [80].

The insulin/insulin-like growth factor (Igf) pathway modulates longevity in multiple species [177]. The first insights into the role of the insulin pathway in longevity came from* C. elegans*. Mutant worms with reduced insulin signaling (mutation in insulin/insulin like receptor (igf),* daf2*) live twice as long as wild-type ones [186]. The longevity effect of the daf2 gene mutation is mediated through* daf16*, the* C. elegans* homologue of transcriptional factor FOXO. The Igf pathway negatively regulates the downstream acting FOXO transcriptional factor [187]. Knocking down the expression of autophagy genes (atg5, atg12, or bec1) abolishes the longevity effect of reduced insulin signaling in* daf2* mutants. It is worth noting that deletion of bec1 also reduces life span in wild-type worms [188].

*Drosophila* mutants with decreased insulin signaling (mutation in Insulin like receptor (InR) or in insulin receptor substrate* chico*) exhibit slow ageing and increased life span [189, 190]. Similar to* C. elegans* Igf mutants, these mutants also require FOXO for life span extension [191, 192]. Phosphorylation of FOXO by activated Igf prevents its nuclear localization and leads to the transcriptional downregulation of FOXO target genes. FOXO mediates the activation of pathways that inhibit growth and promote stress response [193]. It has been shown that FOXO induces autophagy in* Drosophila* larvae [103]. Furthermore, specific activation of FOXO in head fat body increases life span and oxidative stress tolerance. This localized overexpression of FOXO decreases systemic insulin signaling and it is correlated with a decrease in expression of dilp 2 (insulin-like peptide 2) in neurons [193]. Further studies show that reduced insulin signaling causes transcriptional repression of dawdle, an activin-like ligand in the TGF-beta super family, through FOXO, which in turn activates autophagy, thereby maintaining protein homeostasis. This study also shows that overexpression of Atg8a in muscle is also sufficient for life span extension in* Drosophila* [194].

Progressive muscle degeneration is associated with ageing and this precedes other age-related pathologies across species. However, the mechanism underlying muscle ageing is not completely understood. Muscle degeneration is associated with the accumulation of ubiquitinated protein aggregates, which are also positive for Ref(2)P in* Drosophila*. Overexpression of FOXO, or its target 4E-BP, in muscle prevents protein accumulation and increases muscle function via autophagy in* Drosophila*. Overexpression of FOXO increases Atg gene expression in muscle. RNAi-mediated knockdown of Atg7 to about half in FOXO overexpression backgrounds partially increases protein accumulation, suggesting that the effects of FOXO overexpression require autophagy. Moreover, the increase in muscle function by FOXO/4E-BP overexpression is sufficient to extend life span. FOXO/4E-BP overexpression in muscles regulates organism-wide protein homeostasis by reducing feeding and also by decreasing the release of insulin-like growth factors from neurosecretory cells in the brain [195].

JNK signaling plays a major role in regulating ageing in* Drosophila*. Activation of JNK signaling increases tolerance to oxidative stress and extends life span [196]. Life span extension upon JNK activation is also mediated through FOXO. Flies with reduced FOXO activity fail to extend life span and exhibit reduced tolerance to oxidative stress even upon JNK activation. The JNK pathway antagonizes the ISS pathway and promotes the translocation of FOXO to the nucleus [197]. Nuclear translocation of FOXO results in the transcription of autophagy genes [103]. JNK/FOXO reduces Igf activity systemically by reducing dilp2 expression in neuroendocrine cells [197]. JNK-mediated protection from oxidative stress is abolished in flies with compromised autophagy, and the induction of JNK signaling may activate autophagy through FOXO [198].

Spermidine, a naturally occurring polyamine, increases life span in multiple species. Levels of polyamines have been shown to decrease during ageing [199]. Dietary supplementation of spermidine induces autophagy and extends life span in* Drosophila*, and spermidine-mediated longevity is abrogated in flies which lack Atg7 [199]. Moreover, spermidine triggered autophagy inhibits the age-associated cognitive impairment in* Drosophila* [200]. Spermidine regulates ageing most likely by epigenetically regulating autophagy. Spermidine inhibits histone acetyltransferases (HAT), which in turn cause a global deacetylation of histone H3 and activation of autophagy in yeast [199]. Interestingly, spermidine treatment may confer oxidative stress resistance both in autophagy-dependent and autophagy-independent ways in* Drosophila* [201].

The TOR pathway modulates ageing in multiple species. Decreased TOR signaling is associated with an increase in life span and increased tolerance to stress. Treatment of* Drosophila* with rapamycin (an inhibitor of TOR) increases life span and tolerance to both nutrient starvation and oxidative stress. Rapamycin-mediated life span extension is abrogated in flies undergoing Atg5 RNAi [202]. Genetic inhibition of TOR also increases life span in flies [203]. This is likely due to the fact that TOR inhibition activates autophagy [5].

Dietary restriction (reduced food intake without malnutrition) has been shown to be an effective intervention to expand lifespan in multiple species, including* Drosophila* [174, 204]. Cellular pathways that mediate the longevity effect of dietary restriction are not fully understood. Studies in* C. elegans* show that autophagy is required for the longevity effect of dietary restriction. When autophagy is compromised (by deleting bec-1 and ce-atg7) in* eat-2* mutants (a genetic model for dietary restriction in* C. elegans*), longevity is blocked [205]. In fact, most longevity pathways have been suggested to converge on autophagy genes in worms [206].

### 5.3. Autophagy and Neurodegeneration

Neurodegenerative diseases encompass a group of progressive disorders characterised by memory loss, cognitive impartment, loss of sensation, and motor dysfunctions. The cellular hallmark of neurodegenerative disease is the presence of ubiquitinated protein aggregates and neuronal cell death [207]. Several lines of evidence connect autophagy with neurodegeneration. Autophagy maintains cellular homeostasis by removing aggregated proteins and damaged organelles. This process is the most critical in neurons, because neurons do not divide and cannot get rid of protein aggregates through self-replication or self-renewal [208].

One of the risk factors for neurodegenerative diseases is ageing. Ageing is associated with decreased autophagy [208]. The connection between autophagy, ageing, and neurodegeneration is described in detail in Section 5.2.

Several neurodegenerative disease models have been developed in* Drosophila*, based on overexpressing wild type or mutant versions of human disease proteins. These disease models also provide insights into the role of autophagy in the context of neurodegeneration [207].

The overexpression of a human huntingtin protein containing a 120-amino acid long polyQ expansion causes age-dependent degeneration in* Drosophila* compound eye [209]. Treatment of these flies with rapamycin reduces retinal degeneration in an autophagy-dependent manner, similar to results observed in mouse and cell culture models of HD [210]. Further studies showed that the beneficial effect of rapamycin was not restricted to huntingtin disease. Rapamycin treatment alleviates neurodegenerative phenotypes in* Drosophila* nonhuntingtin polyglutamine, polyalanine, and tau disease models [211]. Induction of autophagy by rapamycin is conserved from yeast to mammals. A high-throughput drug screen identified three novel drugs, which induce autophagy independent of TOR. These small molecules reduce the number of protein aggregates and cytotoxicity, both in cellular and* Drosophila* models of neurodegenerative disease [212, 213]. Overexpression of Rab5 also ameliorates huntingtin-induced cell death in* Drosophila*, potentially by the formation of a Rab5 complex with Beclin-1 and Vps34, leading to enhanced autophagosome formation [214].

An independent study documented that hyperactivation of the TOR pathway suppresses autophagy and leads to neuronal cell death. Overexpression of Rheb, an activator of TOR, causes age- and light-dependent degeneration in the* Drosophila* retina. This was likely due to autophagy suppression, as autophagy induction by Atg1 was sufficient to rescue retinal degeneration. Similarly, overexpression of Atg1 or genetic inhibition of TOR by overexpressing TSC1/2 alleviates the neurodegenerative phenotype in* Drosophila* HD and phospholipase C- (norpA-) mediated retinal degeneration models. This study suggests that neurodegenerative symptoms observed in these flies are due to TOR-dependent suppression of autophagy, and not due to the effect of TOR on cell growth [215].

Puromycin-sensitive aminopeptidase (PSA) is the only cytosolic enzyme capable of degrading polyQ sequences. PSA has been shown to be involved in neurodegeneration in* Drosophila*, mice, and cell culture models of poly Q diseases. Overexpression of PSA inhibits polyQ toxicity, whereas inhibiting PSA expression enhances poly Q toxicity in* Drosophila* models of poly Q diseases. PSA was suggested to reduce polyQ toxicity by activating autophagy and subsequent clearance of toxic aggregates, but how it may promote autophagy is still unknown [216].

Results of a genetic modifier screen aimed at the identification of genes involved in Ataxin3 toxicity in* Drosophila* found numerous candidates. A subset of the suppressors was proposed to act either by enhancing autophagy-mediated clearance of protein aggregates or by inhibiting autophagy to prevent autophagy-mediated cell loss. This study also pointed out that only the pathogenic form of ataxin3, and not wild type ataxin, induces autophagy [217].

Induction of autophagy does not rescue neurodegeneration caused by the polyglutamine-containing atrophin in* Drosophila* DRPLA (dentatorubropallidoluysian atrophy) model. The neurodegenerative phenotype is characterized by the accumulation of autophagic vacuoles in degenerating neurons and glia. Inhibiting autophagy by Atg5 RNAi or using an Atg1 null mutant enhances neurodegenerative phenotypes. However, both pharmaceutical and genetic inductions of autophagy failed to rescue neurodegeneration. Ultrastructural analysis showed the presence of abnormally large autolysosomes with impaired degradation of the contents. Thus, the beneficial effect of autophagy may be suppressed by lysosomal dysfunction in this case [218]. Transcriptional profiling identified that atrophin reduces the expression of fat, a tumor suppressor protein. Fat, and Hippo kinase acting downstream of it, may protect the neuron by activating autophagy [219]. Although the exact mechanisms of neuroprotection by the Fat/Hippo pathway are not fully understood, authors of these studies suggested two plausible mechanisms: (1) Hippo may activate autophagy by inhibiting TOR, or (2) Hippo might enhance autophagy through its interaction with Atg8a [220].

An immunoelectron microscopy study identified the accumulation of abnormal autophagic vacuoles (AV) in human AD brain [221]. In line with that, overexpression of A*β*42 (the byproduct of APP proteolysis, a major component of Abeta inclusion in AD) results in age-dependent dysfunction of autophagy at a lysosomal stage in* Drosophila* [222]. This is characterised by the accumulation of abnormal autophagic vacuoles in the brain. The leakage of these vacuoles causes the acidification of cytosol, and further damage to membranes and organelles eventually leads to neuronal cell death. In contrast, overexpression of A*β*40, another byproduct of APP proteolysis, does not cause autophagy dysfunction or neuronal abnormality. This differential neurotoxicity raises the possibility that A*β*40 is degraded by autophagy. Interestingly, inhibition of autophagy partially rescues the neurodegenerative phenotype and activation of autophagy exuberates symptoms in A*β*42* Drosophila* models. The authors of this study suggest that autophagy may act as a prosurvival pathway in early stages of the disease, and as a prodeath pathway in later stages [222].

Studies in* Drosophila* provide potential mechanistic links between UPS and autophagy. Autophagy is induced as a compensatory mechanism during proteasome dysfunction. This compensatory induction is dependent on histone deacetylase 6 (HDAC6), a microtubule-associated deacetylase that interacts with polyubiquitinated proteins. Autophagy is induced in temperature sensitive proteasome mutant flies, and also in response to UPS impairment in* Drosophila* SBMA (spinobulbar muscular atrophy (SBMA)) models. Overexpression of HDAC6 was shown to rescue degenerative phenotypes associated with UPS dysfunction in an autophagy-dependent manner in these flies. Furthermore, HDAC6 overexpression rescues neurodegenerative phenotypes observed in* Drosophila* Ataxia and Abeta models. The rescuing effect of HDAC was again abolished in flies with impaired autophagy [223].

Studies in* Drosophila* have also contributed to our understanding of the link between endocytosis and neurodegeneration and its relation to autophagy. Mutations in the Endosomal Sorting Complex Required for Transport- (ESCRT-) III subunit CHMP2B are associated with FTD (frontotemporal dementia) and ALS (amyotrophic lateral sclerosis). These diseases are characterized by the presence of ubiquitinated protein aggregates, which are positive for p62/SQSTM1. The ESCRT complex is involved in the recognition and sorting of ubiquitinated endocytosed integral membrane proteins into the intraluminal vesicles of the multivesicular body (MVB) and is required for their subsequent degradation in lysosomes. Autophagic degradation is inhibited in cells overexpressing CHMP2B and in cells or* Drosophila* lacking ESCRT function. Reduced ESCRT function impairs the clearance of mutant huntingtin protein in cell and* Drosophila* models of HD diseases. These studies show that the functional MVB pathway is important for proper autophagic function [51, 224, 225].

## 6. Selective Autophagy in* Drosophila*

The Atg8 family proteins are required for the expansion of the phagophore membrane and also participate in cargo recognition and recruitment to the forming autophagosome. These ubiquitin-like (UBL) proteins are conjugated to phosphatidylethanolamine (PE) and are found both on the inner and outer sides of the autophagosome membrane. The Atg8 family proteins including LC3 (microtubule-associated protein 1 light chain 3) lie at the heart of selective autophagy, through their binding to selective autophagy receptors. Six receptors have been identified in mammals so far: p62/SQSTM1/SQSTM1, NBR1, NDP52, Nix, optineurin, and Stbd1 [226–228]. These proteins contain a LIR/LRS (LC3-interacting region/LC3 recognition sequence) motif and have been shown to interact with LC3 family proteins [198, 199].

### 6.1. Selective Autophagy Receptors in* Drosophila*

In* Drosophila*, only two selective autophagy receptors have been described so far: Ref(2)P, the homologue of mammalian p62/SQSTM1/SQSTM1, and blue cheese, the homologue of mammalian Alfy. p62/SQSTM1/SQSTM1 is the first and best understood selective autophagy cargo receptor. It is a multifunctional protein, performing a variety of functions in the cell [229, 230]. Human p62/SQSTM1 is 440 amino acids long and contains several functional motifs [229]. A Phox and Bem1p (PB1) domain is located at the N-terminus and is necessary for the multimerisation of the protein, as well as its interaction with a range of kinases (MEKK3, MEK5, ERK, PKC*ζ*, PKC*λ*/t, and another autophagy receptor, NBR1) [229]. Following the PB1 domain is a ZZ zinc-finger domain, which interacts with the serine-threonine kinase receptor-interacting protein 1 (RIP1) [230]. Importantly, p62/SQSTM1 contains an LC3 interacting LIR/LRS motif, and a kelch-like ECH-associated protein 1 (KEAP1) interacting region (KIR) motif, which interacts with KEAP1 [231–233]. At its C-terminus, p62/SQSTM1 retains an ubiquitin-associated (UBA) domain, required for binding monomeric and multimeric ubiquitin [229].

**Figure 4:**
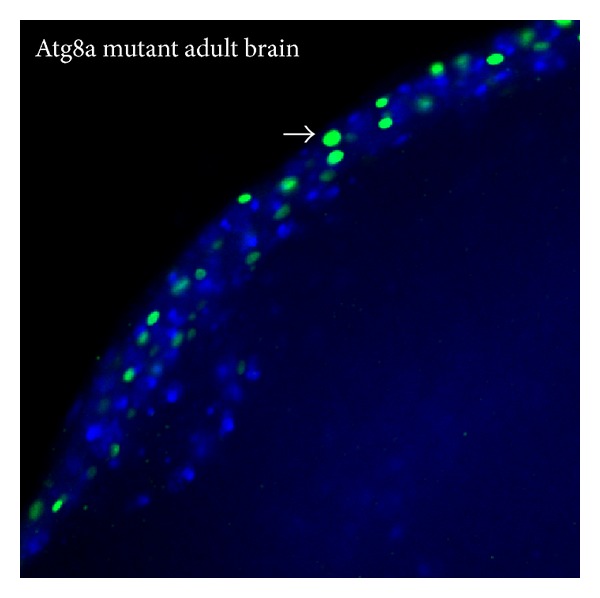
Ref(2)P accumulates in the brain of Atg8a mutant adult flies. Confocal micrograph of a mid-section of the optic lobe in the brain of an Atg8a mutant adult fly. The tissue is stained for Ref(2)P (green, arrow highlights an aggregate) and DNA (blue).

p62/SQSTM1 binds to polyubiquitinated proteins and crosslinks these to the growing phagophore via Atg8/LC3 binding. A reduction in p62/SQSTM1 expression increases huntingtin-induced cell death in HD cell culture models [231, 234]. Autophagy deficient mice lacking p62/SQSTM1 failed to form ubiquitin positive aggregates, indicating that p62/SQSTM1 is important for aggregate formation [235]. The* Drosophila* p62/SQSTM1 homologue, Refractory to Sigma P (Ref(2)P), is 599 amino acids long and also contains an N-terminal PB1 domain, a ZZ-type zinc-finger domain, and a C-terminal UBA domain [236]. Similar to p62/SQSTM1, Ref(2)P is accumulated when autophagy is impaired and it has been found within protein aggregates in autophagy deficient* Drosophila* and in* Drosophila* neurodegenerative models [236] (Figure 4). It makes use of its PB1 domain to multimerise and is able to bind ubiquitin molecules via its UBA domain [237]. Ref(2)P also harbours a LIR motif between residues 451–458 (DPEWQLID) [237, 238], which fits well with the revised LIR motif sequence, proposed by Johansen and Lamark, which could be written as D/E-D/E-D/E-W/F/Y-X-X-L/I/V [229]. Ref(2)P has recently been established as a selective autophagy substrate in* Drosophila* as well [75]. Moreover, it has a putative KIR motif and its interaction with both Keap1 and Atg8a appears to be conserved, too [73, 238, 239].

S6 kinase is a central regulator of autophagy and cell growth. TOR activation suppresses autophagy and leads to the phosphorylation of S6K. S6K was long considered as an autophagy inhibitor, a fact now contested, as S6K is found to be required for starvation-induced autophagy [62, 240]. Consistent with these observations, loss in S6K significantly increased the number (but not the size) of Ref(2)P aggregates in* Drosophila* larval fat body cells [57].

A novel role of Ref(2)P was reported in* Drosophila* haemocytes. Alongside Atg1, Ref(2)P-mediated selective autophagy was shown to be indispensable for cellular remodelling of the haemocyte cortex [241, 242]. Arresting autophagy with 3-methyladenine (3MA) or knocking down other Atg genes (Atg4, Atg6, Atg7, Atg8a, and Atg9) all produced a similar phenotype. Taken together, the above information demonstrates that Ref(2)P has a wide spectrum of cellular functions, like its human p62/SQSTM1 homologue, whose functions require further elucidation.

Loss of function mutation in* Drosophila* blue cheese gene (bchs) results in an age-dependent accumulation of ubiquitinated protein aggregates and amyloid precursor-like proteins and reduces life span. Abnormal central nervous system morphology and size were also documented in bchs mutants [243]. The ubiquitinated protein aggregates in bchs mutants are positive for Ref(2)P [244]. Alfy, the human homologue of* Drosophila* blue cheese, is involved in the selective disposal of ubiquitinated protein aggregates. Alfy is a large, 3527 amino acid long protein, which contains a variety of functional domains, including a FYVE domain suggesting an affinity for PI(3)-P rich endosomes. Instead, Alfy has been found to localise mostly to the nuclear envelope, but it translocates to autophagic membranes and ubiquitin-rich aggregates under strenuous cellular conditions [245]. Alfy-mediated aggrephagy makes use of p62/SQSTM1, the human homologue of* Drosophila* Ref(2)P. Alfy, together with p62/SQSTM1, may crosslink ubiquitinated protein aggregates with the core autophagy machinery for disposal, highlighting the importance of this so-called aggrephagy in neuronal homeostasis [246]. A genetic modifier screen based on the overexpression of blue cheese in* Drosophila* eye has linked lysosomal dysfunction to altered ubiquitin profiles and reduced life span and shows the genetic interaction between certain genes and blue cheese [247, 248]. Alfy has been shown to play a role in the removal of high polyQ-containing mutant huntingtin [246]. Blue cheese overexpression has been observed to rescue morphological and functional qualities in fly eyes expressing a polyQ127 transgene. Recent work by the Simonsen and Finley groups has established a link between overexpression of blue cheese C-terminal region and a general improvement of neurodegenerative phenotypes* in vivo* [246].

### 6.2. Selective Autophagy and Chaperone Assisted Autophagy

Chaperone-assisted autophagy (CAA) differs from macroautophagy in the method of cargo transport, which is mediated by chaperones in CAA, rather than via autophagosomes. However, there is a level of interplay between CAA chaperones and selective autophagy adaptor proteins, which uncovers a hybrid degradative solution, termed Chaperone-assisted selective autophagy (CASA). The* Drosophila* melanogaster cochaperone Starvin (Stv) interacts with ubiquitin adaptor Ref(2)P and ubiquitin ligase CHIP in order to coordinate the activity of Hsc70 and HspB8. This CASA complex is behind the selective degradation of damaged components in muscle Z disks. Loss of CASA function has been associated with progressive muscle weakness and general myopathies in flies, mice, and men [249, 250]. High molecular mass ubiquitin conjugates have been observed in mouse muscle tissue with a concomitant increase in the level of BAG-3 (mammalian ortholog of Starvin), as a result of repetitive tetanic contraction. These conjugates were observed to form microaggregates, which partially colocalised with LC3, suggesting an involvement of autophagosomal engulfment, as part of muscle protein degradation [249]. It is possible that selective macroautophagy and selective chaperone-assisted autophagy cooperate, in order to maintain a healthy protein landscape at tissue level.

### 6.3. Mitophagy

Mitophagy (selective autophagic degradation of damage impaired mitochondria) has been recently described in yeast and mammals [251]. Atg8/LC3 was observed to interact with mitochondrial membrane proteins via its LIR motif, such as the yeast Atg32 [252] and the mammalian NIP3-like protein NIX [253, 254]. The mechanism behind mitophagy is tightly connected to the fusion/fission behaviour of the mitochondrial network. A bioenergetically impaired mitochondrion is prevented from fusing back into the network, by the proteasomal degradation of the profusion factor mitofusin, Mfn, also known as marf in* Drosophila*. This behaviour is facilitated by the E3 ligase Parkin, recruited to the outer mitochondrial membrane (OMM) by PTEN-induced putative kinase protein I (PINK1) as a result of a loss in membrane potential [255, 256]. Parkin is thought to target various OMM substrates such as Mfn: ubiquitinating them and targeting them for proteasomal degradation [257]. Fusion incompetent mitochondrial organelles are then removed by selective autophagy [251]. Mutations of Parkin and Pink1 are associated with familial forms of Parkinson's disease (PD). Most of our understanding of Pink1 and Parkin function comes from* Drosophila*. Pink1 or Parkin null mutants exhibit muscle degeneration, male sterility, reduced life span, and an abnormal mitochondrial morphology [258–260]. Overexpression of the mitochondrial fission inducer Drp1, or knocking down the expression of mitochondrial fusion inducers mfn or opa1 rescues the degenerative phenotypes in Pink1 and Parkin mutants. This suggests that Pink1 and Parkin maintain mitochondrial morphology at least in part by preventing mitochondrial fusion or by enhancing mitochondrial fission [261]. Pink1 and Parkin have been shown to be involved in mitophagy in mammalian cells [255]. Genetic analysis in* Drosophila* showed that Pink1 acts upstream of Parkin [258]. Recruitment of Parkin to mitochondria causes the ubiquitination of mfn in a Pink1-dependent manner. These studies indicate that both Pink1 and Parkin are involved in the removal of dysfunctional mitochondria, and loss of Pink1 or Parkin led to the accumulation of abnormal mitochondria, which causes oxidative stress and neurodegeneration [262, 263].

Recent work by Vincow et al. and colleagues suggests that mitophagy may be the result of an interplay between several processes [264]. Overall mitochondrial protein turnover in parkin null* Drosophila* was similar to that in Atg7 deficient mutants. By contrast, the turnover of respiratory chain (RC) subunits showed greater impairment with relation to parkin loss, than in Atg7 mutants. RC subunit turnover was also selectively impaired in PINK1 mutants [264]. Given the various degrees of mitochondrial protein turnover impairment in response to a deficit in either proteasom- associated factors or selective autophagy regulators, two theories attempt to pinpoint the pathways involved in mitophagy. One model revolves around the chaperone-mediated extraction of mitochondrial proteins [265]. Another possible model involves mitochondria-derived vesicles, which carry selected cargo directly to the lysosome, in an autophagy-independent manner [266]. The latter model has been observed experimentally, whereby vesicles were found to transport a membrane-bound complex IV subunit and contain inner mitochondrial membrane [267].

### 6.4. Novel Selective Autophagy Regulators

Protein ubiquitination is a widespread method for targeting molecules for selective autophagy, including bacteria, mitochondria, and aggregated proteins. As such, ubiquitinating proteins, such as the E1 Atg7, E2 Atg3, and E3 Atg12-Atg5-Atg16 are key regulators of autophagy [226]. Recent work has uncovered the first deubiquitinating enzyme of regulatory importance towards selective autophagy, Usp36 [268]. This protein inhibits selective autophagy in both* Drosophila* and in human cells, while promoting cell growth [269]. Despite phenotypic similarity, Usp36 is not actually part of the TOR pathway [268]. Loss of* Drosophila* Usp36 (*dUsp36*) accompanied the accumulation of aggregated histone H2B (known substrate of Usp36) in cell nuclei, reflecting profound defects of chromatin structure in* dUsp36* mutant cells. Knockdown of dUsp36 led to the accumulation of GFP-LC3 positive vesicles. Anti-LC3B antibody testing revealed an increase in both autophagosome and lysosome formation, inferring total autophagy flux activation in mutant cells and suggesting that USP36 inhibits upstream events of autophagosome initiation [268]. A link was established between p62/SQSTM1-mediated accumulation of ubiquitinated substrates following USP36 inactivation and subsequent induction of autophagy, providing a final piece of evidence that USP36 regulates selective autophagy by inactivating its cognate cargo via deubiquitination [268]. So far, USP36 is the only characterised deubiquitinating enzyme which has been linked to autophagy regulation. Recent studies have identified another two deubiquitinating enzymes, USP19 and USP24, both of which exert negative control on autophagy under normal nutritional conditions [270].

## 7. Conclusion and Future Direction

Studies on morphological aspects and the hormonal regulation of autophagy in insects including* Drosophila* have a long and successful history. More recently, molecular genetics has enabled the functional analysis of autophagy in this complete animal, in which all major tissue types and organs are found and function in many ways similar to our own body. Autophagy studies in* Drosophila* melanogaster have revealed that it has wide-ranging implications in sustaining homeostasis, with possible links to organism development, the immune response, and the removal of cellular damage and waste often associated with ageing and age-related diseases. From the presented literature, it is apparent that there are many unexplored avenues in the mechanisms and regulation of autophagic degradation in* Drosophila*. To better understand its molecular mechanisms, more efforts should be taken to identify selective autophagy receptors which are thought to govern the remarkable degradation specificity seen in certain settings. These studies will be facilitated by recently developed computer software to predict Atg8-family interacting proteins [271]. Manipulating selective autophagy influences the phenotype in a range of neurodegenerative disease models, such as Alzheimer's [272], Huntington's [273], and Parkinson's [274] diseases, which often revolves around the removal of molecules damaged by reactive oxygen species (ROS), or eliminating ROS synthesis sites such as impaired mitochondria. It would therefore be interesting to test whether upregulating autophagy can facilitate effective removal of proteins associated with neurodegenerative pathologies caused by the expression of hyperphosphorylated tau or high polyglutamine length huntingtin. It might be worth investigating the importance of mitophagy in maintaining a healthy cellular environment and resisting stress, particularly with regard to age-related myocardial degeneration, as this is a vastly underexamined area. Finally, the recent discovery of deubiquitinating enzymes as negative regulators of autophagy lays the ground for further study of a novel class of autophagy regulators.
